# Supplementary material for: Saccharomyces boulardii (CNCM I-745) improves intestinal damage in sepsis by remodeling intestinal flora structure
Source: Front Cell Infect Microbiol. 2025 Jul 31;15:1602792. doi: 10.3389/fcimb.2025.1602792 (PMC12350329; doi:10.3389/fcimb.2025.1602792)
Supplement: Supplementary file 1 [file DataSheet1.zip › summary/summary/8_Advanced_analyse/2_PICRUSt2_result/KEGG/Group.AW4_vs_AW0/PICRUSt2_KO_Group.AW4_vs_AW0_diff.pdf]

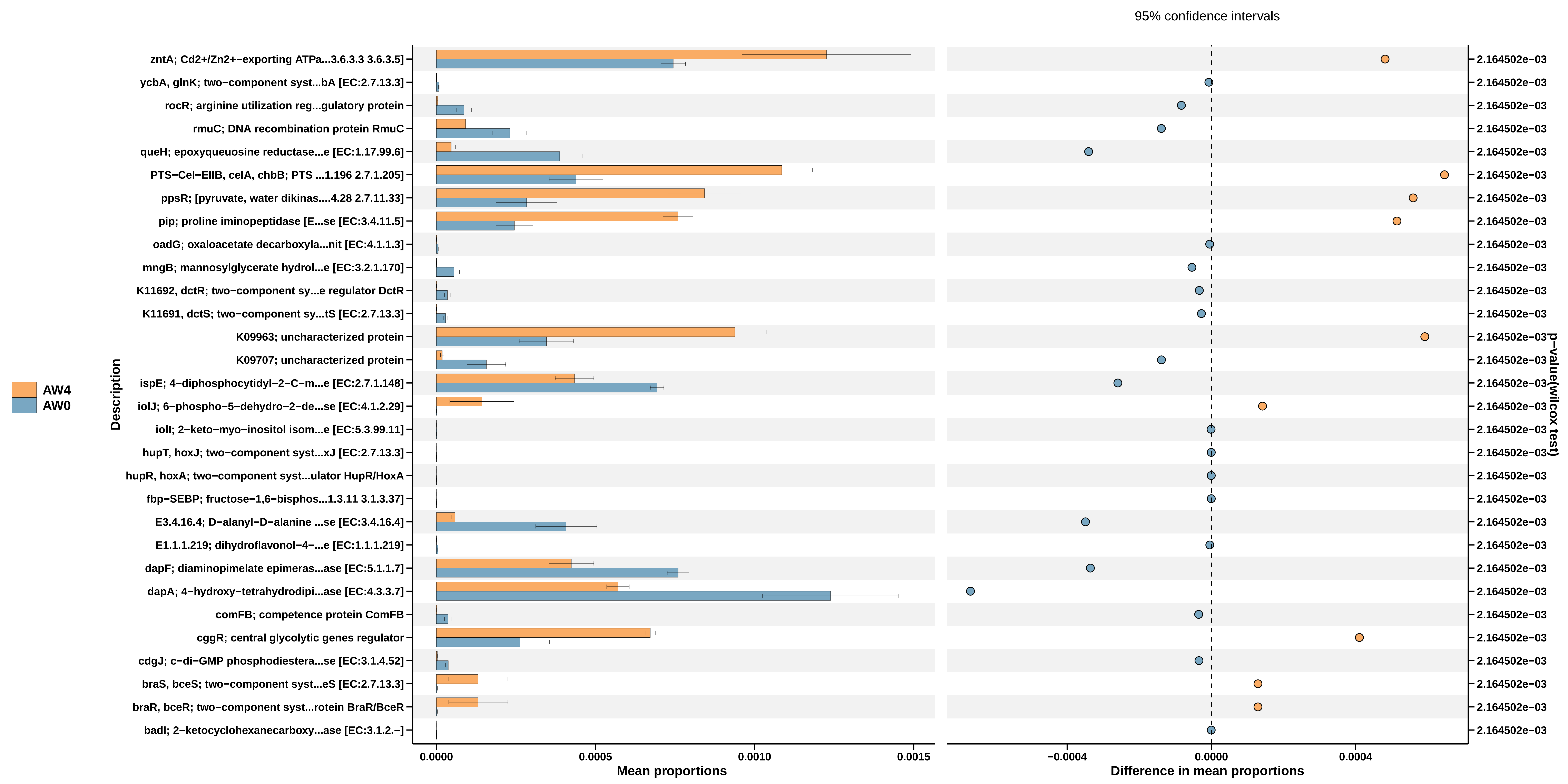

95% confidence intervals
zntA; Cd2+/Zn2+−exporting ATPa...3.6.3.3 3.6.3.5]
ycbA, glnK; two−component syst...bA [EC:2.7.13.3]
rocR; arginine utilization reg...gulatory protein
rmuC; DNA recombination protein RmuC
queH; epoxyqueuosine reductase...e [EC:1.17.99.6]
PTS−Cel−EIIB, celA, chbB; PTS ...1.196 2.7.1.205]
ppsR; [pyruvate, water dikinas....4.28 2.7.11.33]
pip; proline iminopeptidase [E...se [EC:3.4.11.5]
oadG; oxaloacetate decarboxyla...nit [EC:4.1.1.3]
mngB; mannosylglycerate hydrol...e [EC:3.2.1.170]
K11692, dctR; two−component sy...e regulator DctR
K11691, dctS; two−component sy...tS [EC:2.7.13.3]
K09963; uncharacterized protein
K09707; uncharacterized protein
ispE; 4−diphosphocytidyl−2−C−m...e [EC:2.7.1.148]
iolJ; 6−phospho−5−dehydro−2−de...se [EC:4.1.2.29]
iolI; 2−keto−myo−inositol isom...e [EC:5.3.99.11]
hupT, hoxJ; two−component syst...xJ [EC:2.7.13.3]
hupR, hoxA; two−component syst...ulator HupR/HoxA
fbp−SEBP; fructose−1,6−bisphos...1.3.11 3.1.3.37]
E3.4.16.4; D−alanyl−D−alanine ...se [EC:3.4.16.4]
E1.1.1.219; dihydroflavonol−4−...e [EC:1.1.1.219]
dapF; diaminopimelate epimeras...ase [EC:5.1.1.7]
dapA; 4−hydroxy−tetrahydrodipi...ase [EC:4.3.3.7]
comFB; competence protein ComFB
cggR; central glycolytic genes regulator
cdgJ; c−di−GMP phosphodiestera...se [EC:3.1.4.52]
braS, bceS; two−component syst...eS [EC:2.7.13.3]
braR, bceR; two−component syst...rotein BraR/BceR
badI; 2−ketocyclohexanecarboxy...ase [EC:3.1.2.−]
2.164502e−03
Description
AW4
AW0
0.0000
0.0005
0.0010
0.0015
Mean proportions
−0.0004
0.0000
0.0004
Difference in mean proportions
p−value(wilcox test)
